# Portraying Tuberculosis through Western Art, 1000–2000 CE

**DOI:** 10.3201/eid3103.231581

**Published:** 2025-03

**Authors:** Yousra Kherabi, Philippe Charlier

**Affiliations:** Infectious and Tropical Diseases Department, Bichat-Claude Bernard Hospital, Assistance Publique-Hôpitaux de Paris, Université Paris Cité, Paris, France (Y. Kherabi); IAME, INSERM, Université Paris Cité, Paris (Y. Kherabi); Université Paris-Saclay, Montigny-le-Bretonneux, France (P. Charlier); Biologie–Institut de France, Paris (P. Charlier)

**Keywords:** Tuberculosis and other mycobacteria, bacteria, tuberculosis, art, painting, representation, *Mycobacterium tuberculosis*

In 2025, tuberculosis (TB) still maintains its grim distinction as one of the world’s most lethal infectious diseases. *Mycobacterium tuberculosis*, the causative agent, infects one fourth of the global population (*1*). However, this statistic offers only a partial understanding of the disease’s true effect on humanity.

To assess the burden of TB comprehensively, especially in Europe, we must embark on a historical expedition, retracing our steps to an era when this ailment was a cryptic and seemingly incurable enigma (*2*). During that period, society sought to grapple with the mysteries of tuberculosis through artistic expressions. Representation of TB in art underwent an evolution in tandem with the shifting perceptions of the disease.

Termed under various appellations, such as phthisis, a Greek term denoting wasting, or consumption, TB has long been portrayed across a spectrum of cultural domains from literature and music to movies (*3**,**4*). The confluence of visual arts and TB offers a unique vantage point to examine humanity’s enduring confrontation with this disease. In this article, we aim to explore the depiction of TB in Western art across the centuries, shedding light on how it not only reflects a medical journey but also echoes the profound societal shifts accompanying its history.

## Methods

### Definitions

The primary objective of this study was to conduct a comprehensive review of the representation of TB in Western pictorial arts spanning a millennium. To establish a focused framework, we defined a precise chrono-cultural context, centering on Western art created during 1000–2000 CE. Geographically, this context encompasses Europe, the United States, and Canada. Our review included a diverse range of pictorial art forms, including painting, engraving, sculpture, photography, and posters.

### Search Strategy

To identify relevant references for our review, we executed searches across museum databases and national heritage platforms (Appendix). Our search strategy included the use of specific keywords, including tuberculosis, cough, scrofula, consumption, phthisis, king’s evil, disease, and healing. To ensure inclusivity, those search terms were translated into the language of each database.

### Selection of Artworks

From our extensive search, we selected reference artworks that portrayed TB according to previously published iconodiagnosis guidelines (recommendations for the retrospective diagnosis carried out on a work of art representing a human being) (*5*). We excluded pieces of art that did not unequivocally depict the presence of TB. The process of selection ensured the chosen artworks provided clear and discernible representations of TB within the context of our study.

## Results

**Figure 1:**
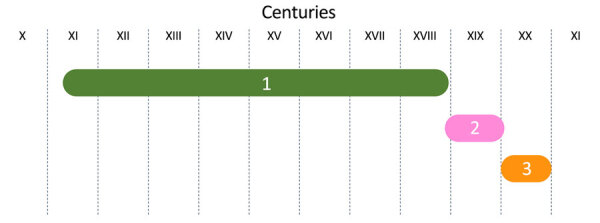
The 3 different periods of consequence in tuberculosis representation in visual art in the Western world, 1000–2000 CE. The first period (10th–18th Centuries) was marked by the magic of “the royal touch.” The second period (19th Century) displayed romanticized representations of tuberculosis. The third period (20th Century) depicted the struggle against an identified microbial enemy: Koch’s bacillus.

We classified the selected works of art into 3 different periods of influence (Figure 1). The first period, from the 10th Century through the 18th Century, was marked by the depiction of thaumaturgic kings (i.e., kings with miraculous healing powers); the most famous wonder was the touching of scrofula. This period was followed by a very short but rich second period that flourished at the start of the Industrial Revolution and was full of paradoxes. The third period covered the 20th Century, which was a period of challenge and struggle against an identified scourge: Koch’s bacillus.

### First Period, 10th–18th Centuries

Throughout history, rulers have sought divine approval to legitimize their reign, a phenomenon integral to the governance of many cultures (*6*). Rulers in Europe in particular claimed the divine right to rule, and the belief of the divine right of kings in Britain and France played a major role in shaping the past millennium (*7*).

The royal touch, an act by the monarch with which they could seemingly heal the sick, probably dates back to Clovis of France (5th Century) or to Philip I (11th Century) in France and to Edward the Confessor in Britain (11th Century) (*7**, **8*). In Shakespeare’s Macbeth, the royal touch is shown as both a medical ritual and a symbol of the monarch’s legitimacy (*9*). Afflicted persons often sought the king’s miraculous cure for scrofula (tuberculous cervical lymphadenitis), often referred to as the King’s Evil. Before the advent of pasteurization, scrofula was predominantly because of the ingestion of dairy products contaminated with *M. bovis* that resulted in local infection of the lymph nodes in proximity to the upper digestive tract (*10*).

**Figure 2:**
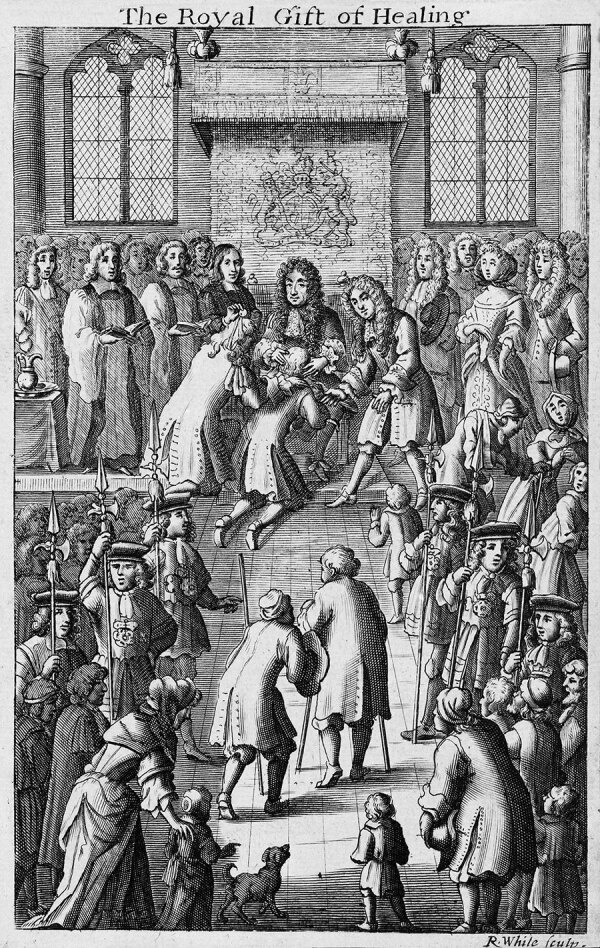
An engraving by Robert White of Charles II touching a patient to cure them of tuberculosis, or the King’s Evil (scrofula), surrounded by courtiers, clergy, and the public. Image from the Wellcome Collection, https://wellcomecollection.org/works/z9hwpcka. Public domain image.

In ceremonies, subjects could approach the king to seek the royal touch, hoping to cure their ailments or diseases (Figure 2). Scrofula would manifest itself with painful and visible sores that could spontaneously go into remission and even resolve, giving the impression of a royally induced cure. Frequently during the 15th–17th Centuries, those subjects were also given a hammered gold coin as a gift picturing the winged standing figure of the Archangel Michael slaying a dragon with a spear (*11*).

### Second Period, 19th Century

**Figure 3:**
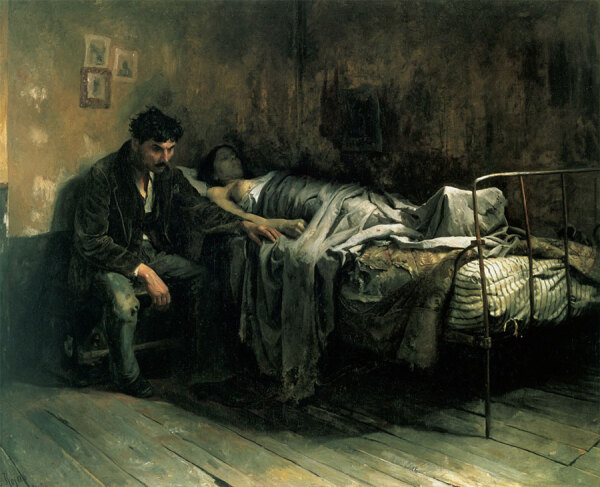
*The Misery*, an oil painting by Cristobal Rojas that depicts tuberculosis as a disease of poverty. 1886. Public domain digital image.

The 19th Century witnessed a rich and paradoxical portrayal of TB in the pictorial arts. As the Industrial Revolution brought about urbanization and widespread poverty, artists began to interpret the disease within this new social context. TB was frequently seen as an ailment of poverty, a theme powerfully encapsulated in Cristobal Rojas’ “The Misery” (Figure 3), a poignant painting of the somber reality of TB in the 19th Century. This painting depicts a young man in a state of despondency next to his wife who has succumbed to the illness amidst the backdrop of squalor. Rojas’ work stands as a vivid reminder of the human cost of TB during a time when the disease was a major cause of death in Europe (*12*). Operas such as Verdi’s “La Traviata” and Puccini’s “La Bohème” also reflected societal views on TB.

**Figure 4:**
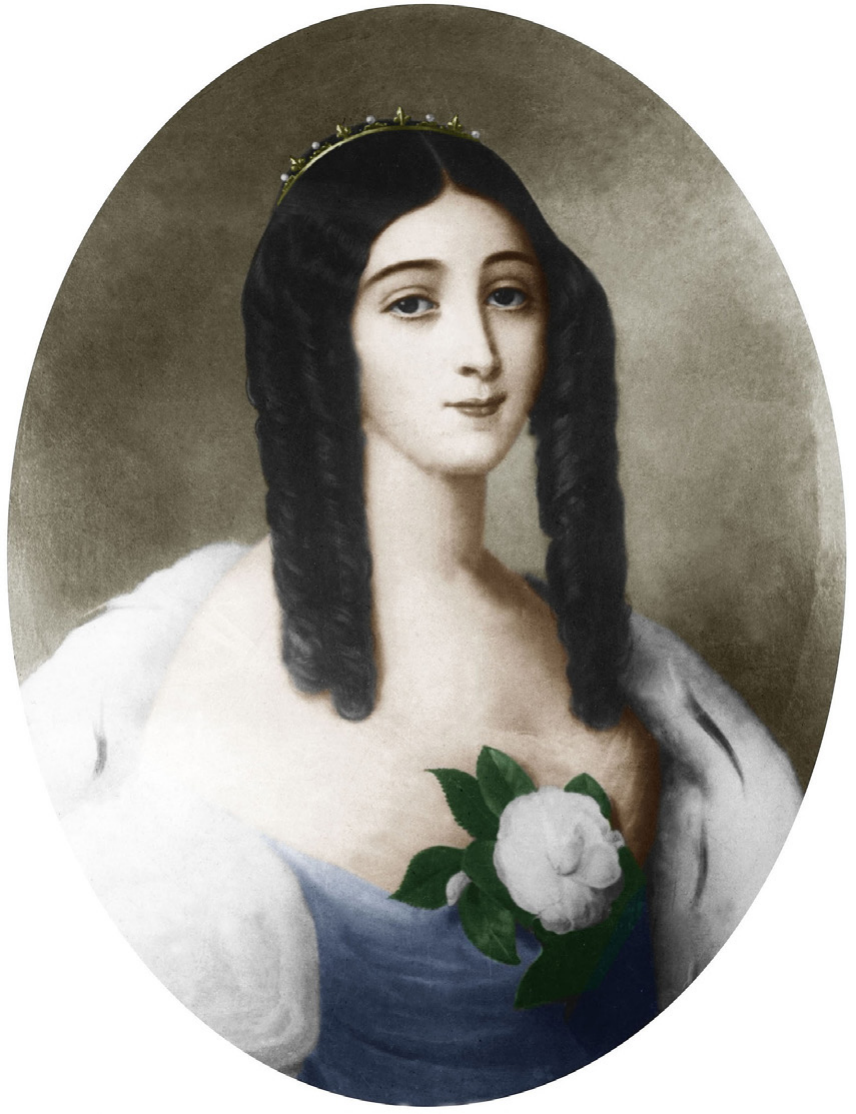
A 19th Century painting of Marie Duplessis by Edouard Viénot. Marie Duplessis was a courtesan with tuberculosis whose beauty contributed to romanticizing the infection. Public domain digital image.

**Figure 5:**
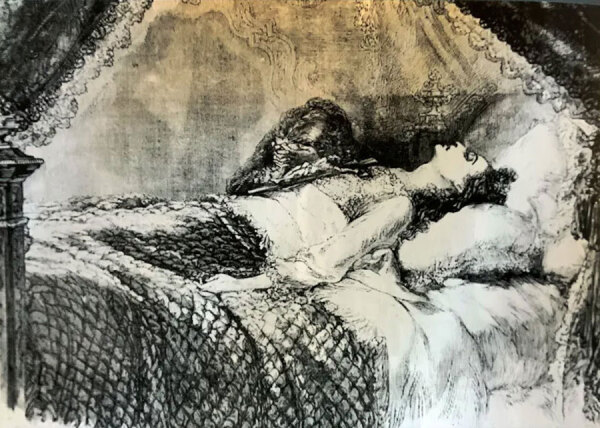
A 19th Century engraving of Marie Duplessis’ death by H. Linton. Created during the romanticized era of tuberculosis history. Public domain digital image.

Concurrently, with the stark realism of tuberculosis’s representation, there emerged a romanticized vision of the disease as a marker of fragile, tragic beauty, a sentiment that became particularly pronounced in the 19th Century (*13*). This idealization of tuberculosis-related frailty was famously captured in the figure of Marie Duplessis, the high-society courtesan whose battle with tuberculosis was immortalized in Alexandre Dumas fils’ “La Dame aux Camélias” (*14*) (Figure 4). Her portrayal as an ethereal beauty, with her pallor, slimness, and radiant eyes, captivated the societal imagination, encapsulating the era’s curious romanticization of consumption. This romanticization was a phenomenon that even Lord Byron alluded to, suggesting that consumption led to a delicate and refined end by enhancing a person’s beauty until the last breath; a jarring contradiction to the harsh reality of the disease (*15*) (Figure 5).

Historically, there was a pervasive belief that TB could accentuate artistic talent. The slight fever and toxemia supposedly enabled artists afflicted with TB to see more clearly and to act more decisively, a notion rooted in Greek medical terms that associated phthisis with heightened mental faculties. This idea was further romanticized during the 19th Century and the physical manifestations of the disease, such as lean limbs and a pallid complexion, were often linked to an aesthetic of the ethereal and the sublime, reinforcing the stereotype of the consumptive artist who produced work of profound emotional and artistic depth (*16*). Poets such as Percy Bysshe Shelley and John Keats transformed their personal battles with TB into metaphors for creativity and passion, exemplifying the concept of “spes phthisica,” where physical decline spurred artistic brilliance (*17*). This romantic notion, although scientifically unfounded, contributed to the mythos of the tortured artist, intertwining the suffering and creativity of figures who, despite their illness, were believed to have harnessed their condition to fuel their artistic genius.

**Figure 6:**
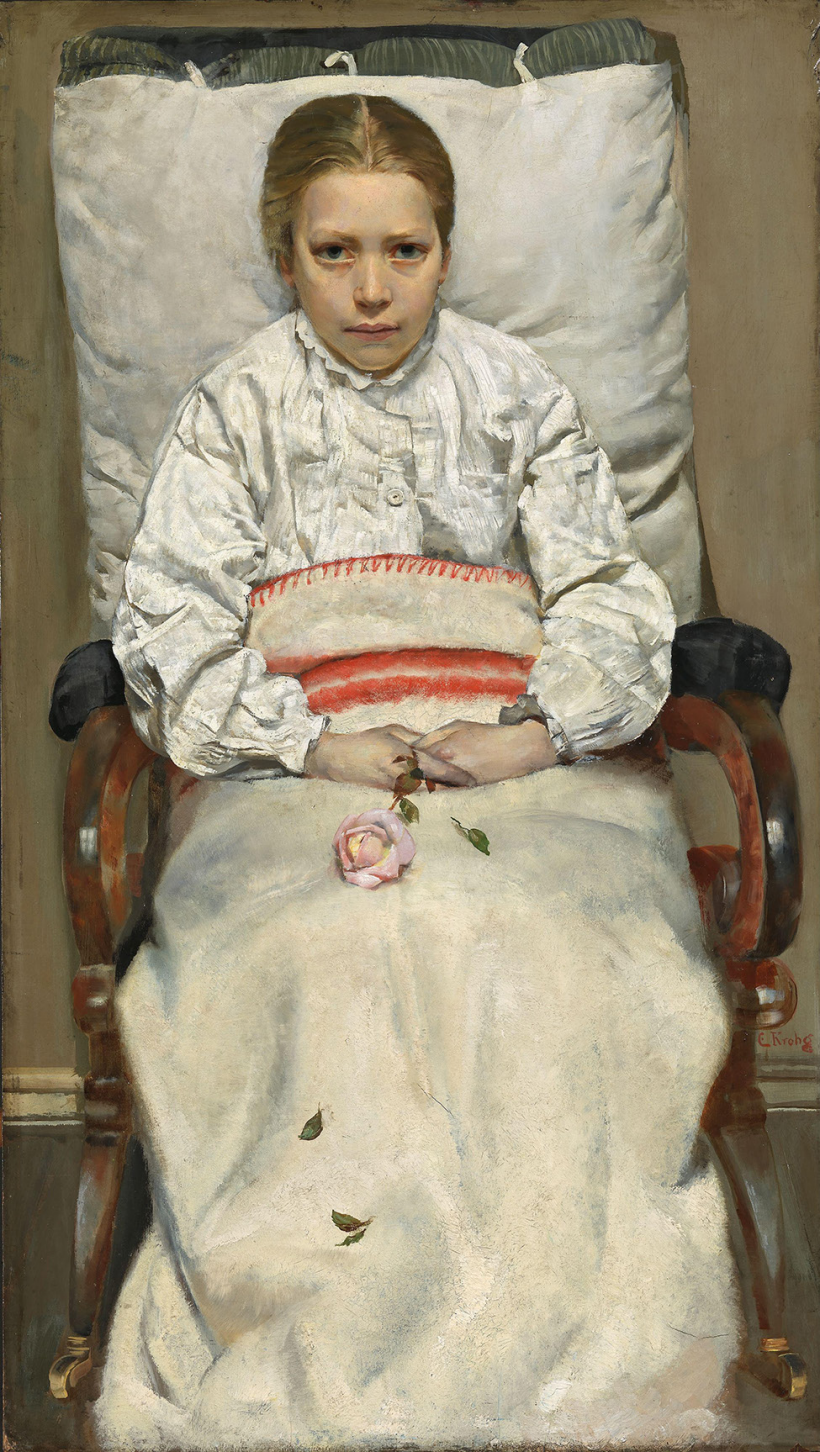
*Sick Girl* is an oil painting by Christian Krohg detailing the familiar heartache experienced by many of losing loved ones to tuberculosis. Public domain digital image.

**Figure 7:**
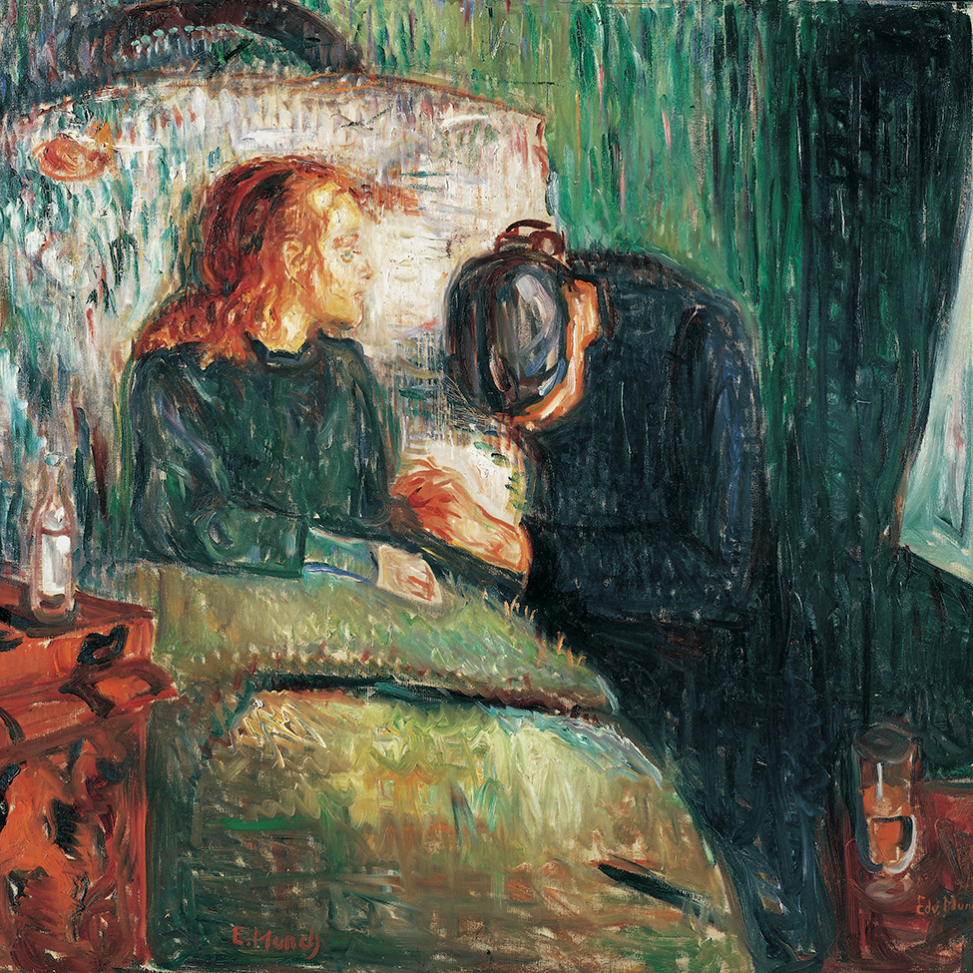
A painting by Edvard Munch, *The Sick Child*, depicts the moments before the death of his sister Sophie from tuberculosis. Munch portrayed his sister Sophie in a chair, in pain, and accompanied by a grieving woman. Image from The Munch Museum/The Munch-Ellingsen Group/Artist Rights Society, NY. Image copyright, Tate, London, 2011. Previously published by Emerging Infectious Diseases (https://wwwnc.cdc.gov/eid/article/17/3/ac-1703_article).

The intimate tragedies of TB within familial circles are profoundly rendered in the works of Christian Krohg and Edvard Munch. Krohg’s “The Sick Girl” from 1880–1881 is a poignant depiction, where the neutral setting and the subject’s simple attire focus the viewer’s attention on the emotional gravity of the scene (Figure 6). Nana, the dying girl, sometimes identified as Krohg’s sister, daughter, or niece, is prominently placed and engages the viewer in a shared space of death, underscored by the emblematic withering rose that symbolizes the fleeting nature of life. This painting, steeped in personal loss with echoes of Nana’s illness and death, is thought to have influenced Edvard Munch. “The Sick Child” by Munch is a series of 6 paintings and various prints created during 1885–1926, depicting the moments before the death of his sister Sophie from tuberculosis (*18*) (Figure 7). Munch revisited this personal trauma in his art for more than 4 decades, portraying Sophie in a chair, in pain, and accompanied by a grieving woman, likely her aunt. Munch’s work symbolizes his own experiences with TB and his feelings of despair and guilt for surviving his sister. Obsessively returning to this theme, he produced numerous versions in different formats by using various models (*19*).

**Figure 8:**
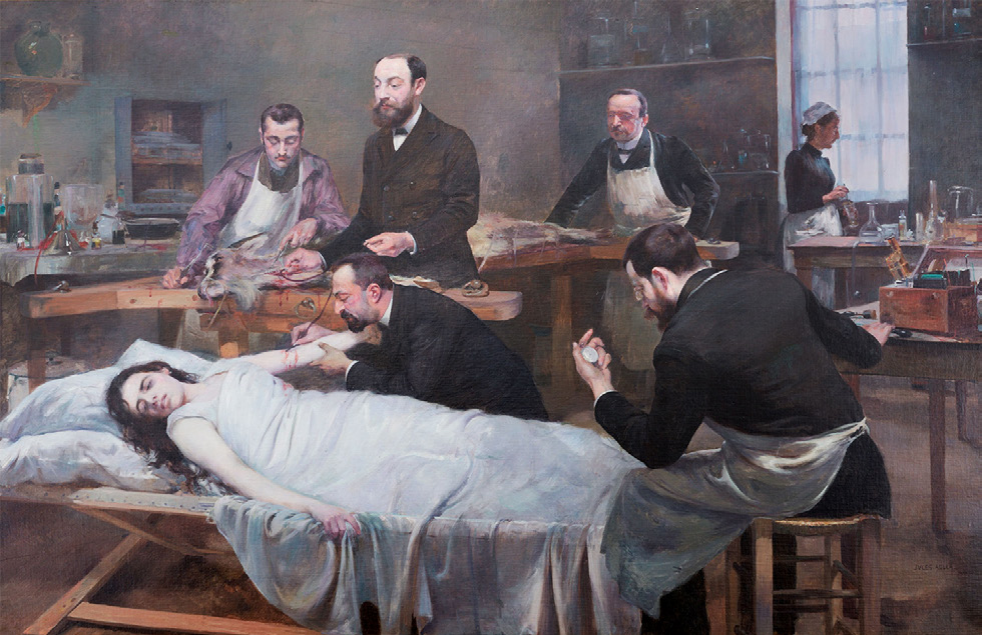
An 1892 painting by Jules Adler, *Transfusion of a Goat’s Blood*. Commissioned by Dr. Samuel Bernheim, a tuberculosis specialist, the painting shows him overseeing a transfusion of goat blood to a patient and demonstrates the engagement of art with medical progress. Copyright © Pittsburgh Post-Gazette, 2010, all rights reserved. Photograph by Alyssa Cwanger, 2006. Previously published by Emerging Infectious Diseases (https://wwwnc.cdc.gov/eid/article/18/8/ac-1808_article).

The turn of the 20th Century was marked by noteworthy medical advances in the fight against tuberculosis as depicted in the fine arts of the period. René Laennec’s innovation of the stethoscope, a revolutionary breakthrough for the diagnosis of TB, was celebrated in art, which showcased the instrument that became synonymous with medical practice. Furthermore, the artistic engagement with medical progress was epitomized by Jules Adler’s “Transfusion of a Goat’s Blood” (Figure 8). Commissioned by Dr. Samuel Bernheim, a renowned physician and TB specialist from Paris, the painting depicts him overseeing a transfusion of goat blood to a patient (*20*, *21**)*. In the painting’s foreground, a woman reclines, enveloped in a pristine white shroud, her complexion ghostly, contrasting with the stark black of her hair and her hand tightly clutches the bed’s edge. Adler’s work reflects the perception of medical practices as grand historical events, thus bridging the realms of art and the history of medicine. This dualistic artistic depiction of TB traverses the societal spectrum from raw reality to idealized romanticism, juxtaposing the gritty struggle against the disease with an almost paradoxical glorification, all amidst a backdrop of critical medical innovation.

### Third Period, 20th Century

The 20th Century marked a shift in the representation of TB because scientific understanding advanced. Robert Koch’s discovery of the TB bacillus in 1882 shattered the romanticized image of the disease. The imagery moved from depicting the consumptive beauty to showcasing TB as an enemy of public health (*17*).

**Figure 9:**
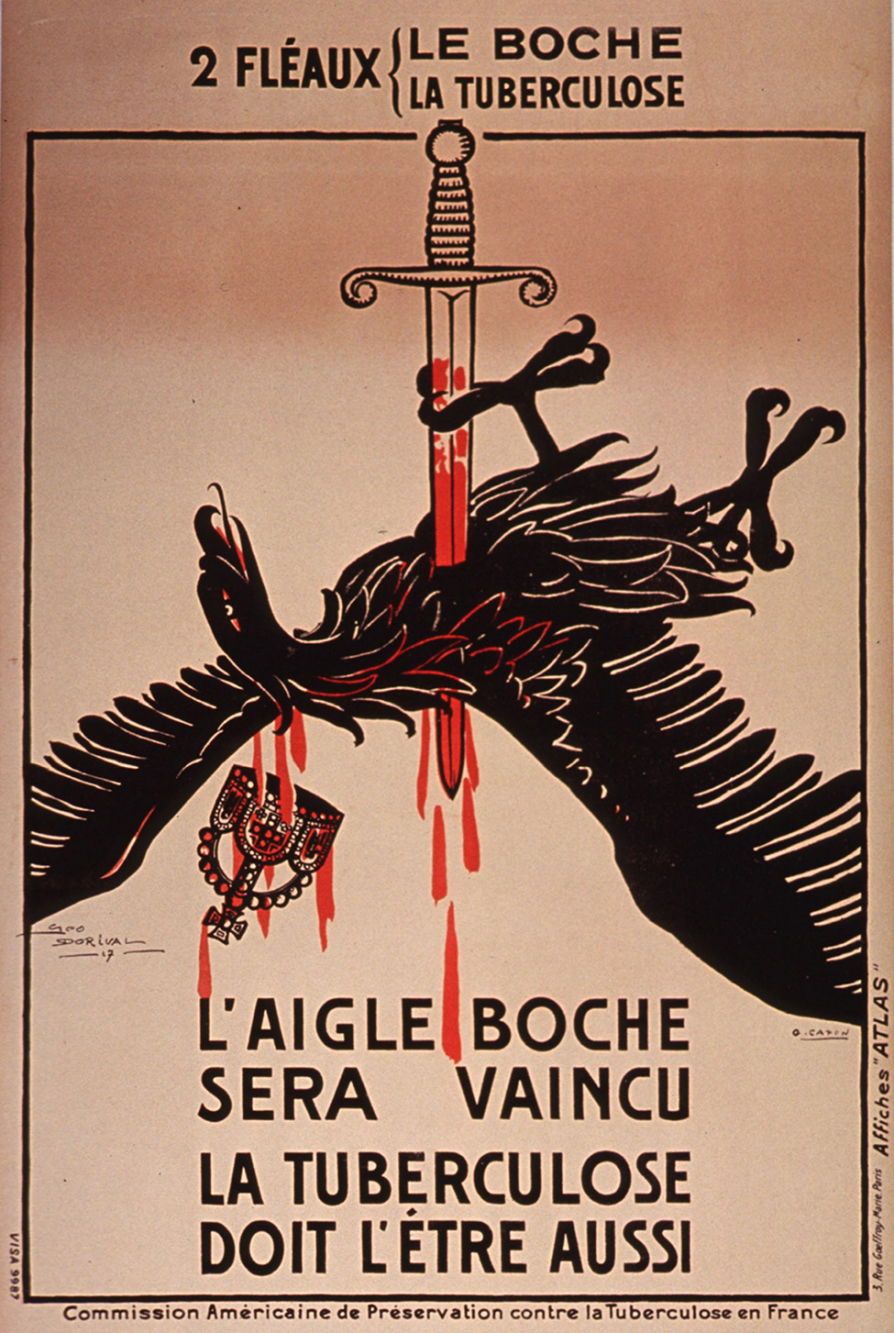
L’aigle Boche sera vaincu: la tuberculose doit l’être aussi, a World War I propaganda poster from France depicting tuberculosis as a national adversary. Public domain digital image.

During World War I, TB was depicted in propaganda posters from France as a national adversary, akin to the German enemy. One propaganda poster shows the German imperial eagle being struck down by a sword, drawing a parallel between the fight against TB and the war against Germany (Figure 9)​​.

The representation of TB in the 20th Century not only documented a medical battle against a microbial foe but also encapsulated the social and political challenges of the era. The fight against TB was not just in hospitals and sanatoriums but also on the front lines of public consciousness, through stamps, posters, and public campaigns, urging a societal call to arms against this persistent threat to human health.

**Figure 10:**
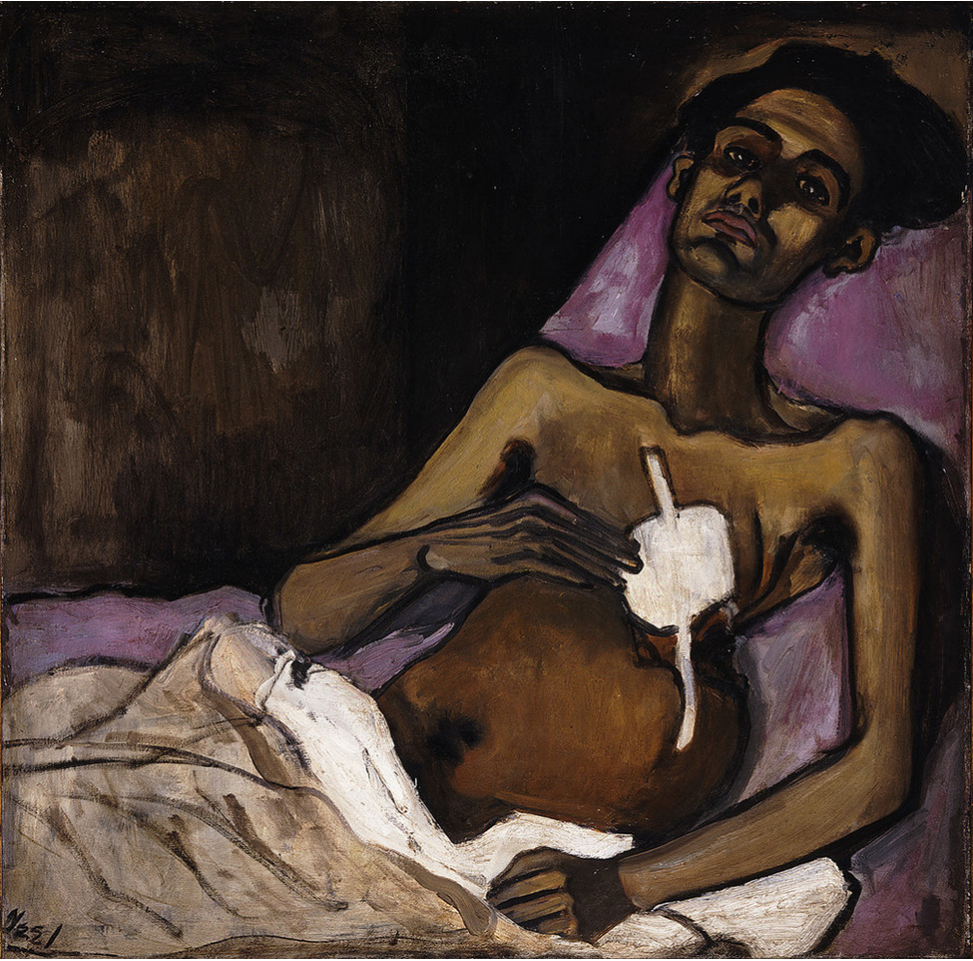
Alice Neels’ 1940 painting *TB Harlem* depicting tuberculosis in New York City, portraying Carlos Negrón after thoracoplasty. Image from the National Museum of Women in the Arts, Gift of Wallace and Wilhelmina Holladay. Copyright © The Estate of Alice Neel/Courtesy of David Zwirner, New York. Previously published by Emerging Infectious Diseases (https://wwwnc.cdc.gov/eid/article/19/3/ac-1903_article).

In the United States, artists such as Alice Neel brought the issue of TB into the context of immigration and the urban experience (*22*). Neel’s 1940 painting “TB Harlem” starkly depicts the reality of TB in New York City, portraying Carlos Negrón with a dignified yet afflicted presence postthoracoplasty (Figure 10). Her unsentimental style emphasizes the physical ravages of the disease through distorted anatomy and dark, heavy outlines. Neel’s work reflects TB’s grim effect in urban settings, particularly within the disadvantaged communities of Harlem.

**Figure 11:**
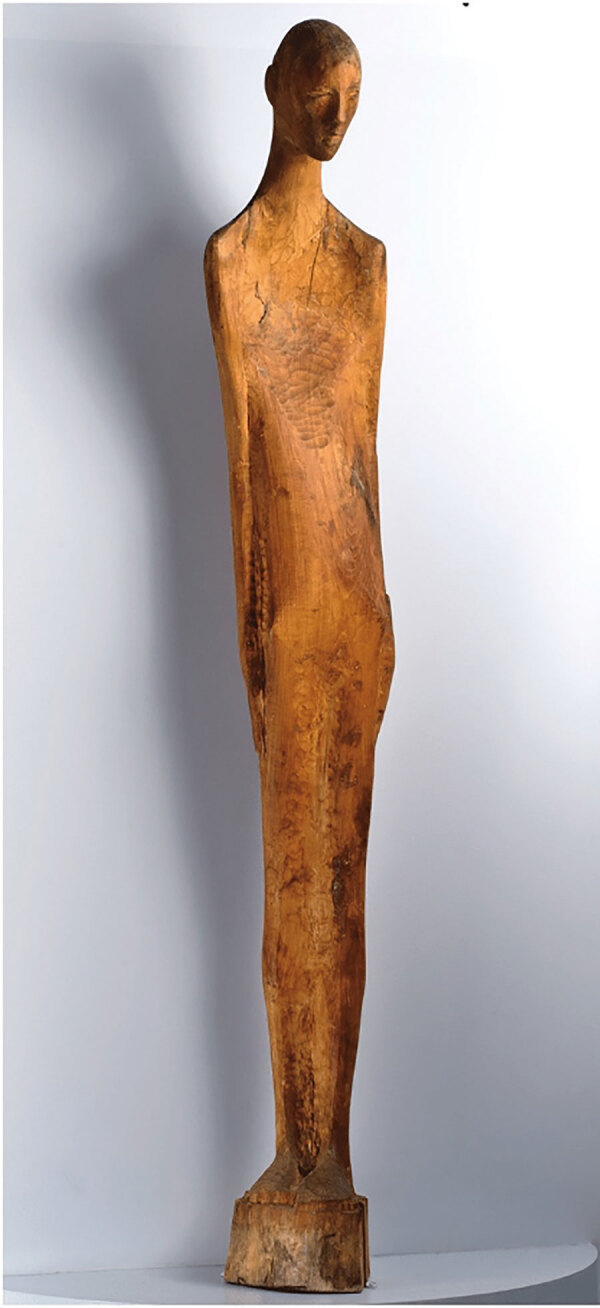
*Recovery*, a life-size wood sculpture by an unnamed tuberculosis patient from an asylum in England, showcasing the patient’s direct experience with the disease as he represented himself with a sunken chest. Copyright © American Visionary Art Museum.

This representation is complemented by “Recovery,” a life-size wood sculpture by an unnamed TB patient from an English asylum, depicting the patient’s own experience with the disease by representing himself with a sunken chest (Figure 11) (*23*). This work underscores the personal narratives of those who endured TB, shifting the focus from mere artistic interpretation to patient-lived reality.

Since the 1980s, TB and HIV have been jointly portrayed in art as twin scourges, reflecting their intertwined epidemiologic effect on global health. Posters and visual campaigns frequently depict them together, symbolizing the compounded vulnerability and the heightened challenge faced by those having both conditions (*24*). This co-representation has served to amplify awareness and galvanize action against the dual public health crises.

## Discussion

Our review has traced the evolution of TB’s portrayal from a mysterious condition affecting all societal levels to a known pathogen targeted by public health initiatives. Throughout the centuries, the representation of TB in Western art has undergone a profound transformation. This artistic journey through TB’s depiction reflects a complex interplay among harsh reality, romantic idealization, and evolving medical understanding, illustrating how deeply TB has been woven into the cultural and artistic fabric of society.

In the organization of this review, we have consciously categorized the artistic representation of TB into 3 distinct periods, a decision driven by our goal to provide clarity and coherence for the reader. Although we acknowledge that the artistic portrayal of TB often transcends strict chronological boundaries and forms a spectrum of evolving expression, this structured approach simplified the complex interplay between art and the disease. By dividing the material into distinct eras, we aimed to highlight the major shifts in perception and representation that paralleled medical advancements and still appreciate the nuanced continuity present in the artistic narrative of this disease.

Artistic portrayals often showed patients as gaunt or skeletal figures, either bedridden or sitting, their bodies weakened or immobilized. However, as the 20th Century progressed, the visual narrative attributed to TB permeated representations of other diseases. Inspired by the TB attributes, the Spanish influenza after 1918 and cancer after the 1950s were portrayed by using similar visual motifs in artistic representations. This shift coincided with a decline in TB because of increasingly effective public health measures, the devastating effect of the Spanish influenza pandemic, and a rise in cancer diagnoses (*25*,*26*).

The 20th Century has also seen the patient’s perspective come to the forefront. Those personal narratives deepen our appreciation for the subjective experience of illness and resonate with contemporary movements in healthcare that emphasize patient-centered perspectives. Those narratives also illustrate how art has not only served as a medium for societal reflection but also provided a therapeutic outlet for persons to process and contend with their conditions (*27*).

**Figure 12:**
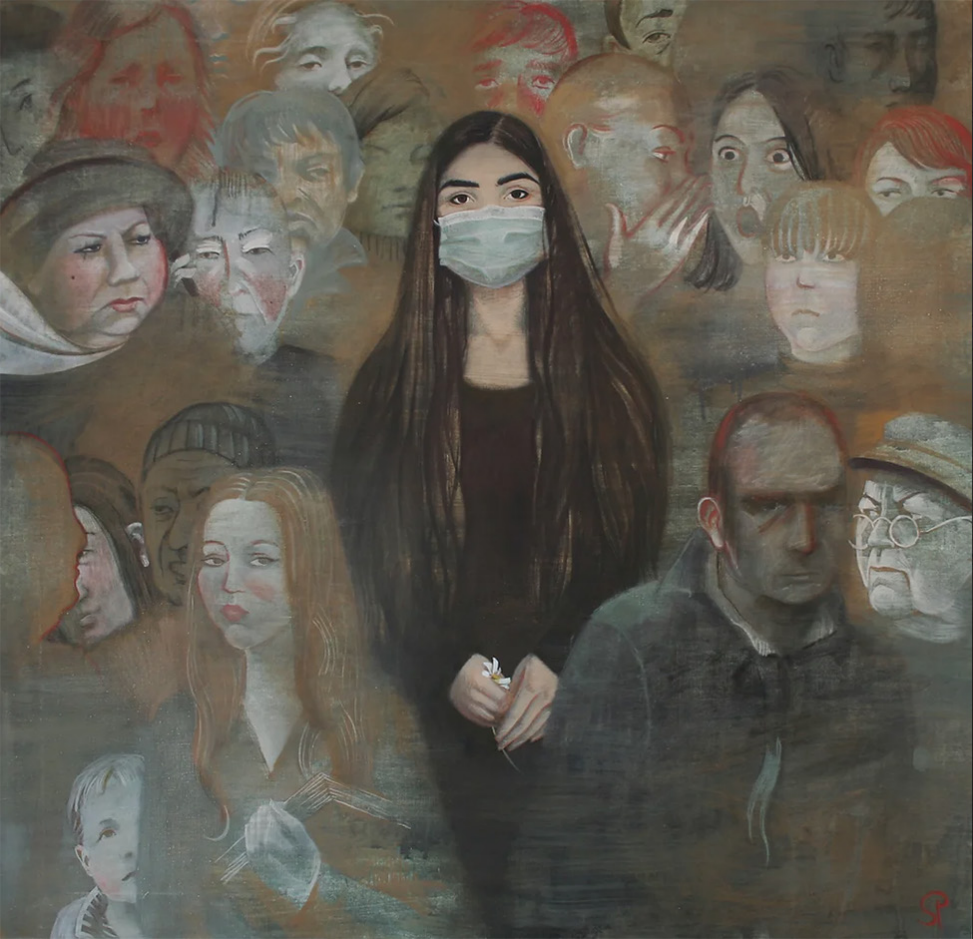
*Don’t Speak* is a painting by Paulina Siniatkina. She painted the piece while battling tuberculosis in 2015 to demonstrate how stigmatizing the infection felt. Copyright © Paulina Siniatkina. Previously published by Emerging Infectious Diseases (https://wwwnc.cdc.gov/eid/article/30/3/AC-3003_article).

Once shrouded in a romanticized veil or seen as an almost divine affliction, TB has now become a stigmatizing disease. In the 21st Century, the works of artists such as Paulina Siniatkina, who, while battling TB herself in 2015, created poignant and powerful paintings during her stay in a TB hospital in Moscow, are particularly illustrative (*28*,*29*) (Figure 12). Her art is a testament to the role of creative expression in coping with illness and stigma, offering both a form of escapism and a way to confront and articulate the reality of living with a chronic condition (*30*).

In conclusion, the intersection of TB and art throughout history highlights the enduring human capacity to find meaning and resilience in the face of suffering. The artistic legacy of TB, from the royal touch to patient-produced artwork, encapsulates a diverse range of human response to this disease. As we continue to grapple with TB in various contexts, art remains a potent form of expression and coping, offering insights into the individual and collective experience of health and disease.

Appendix Additional information on portraying tuberculosis through Western art, 1000–2000 CE.
