# Supplementary material for: Portraying Tuberculosis through Western Art, 1000–2000 CE
Source: Emerg Infect Dis. 2025 Mar;31(3):607–14. doi: 10.3201/eid3103.231581 (PMC11878322; doi:10.3201/eid3103.231581)
Supplement: Appendix — Additional information on portraying tuberculosis through Western art, 1000–2000 CE. [file 23-1581-Techapp-s1.pdf]

Article DOI: https://doi.org/10.3201/eid3103.231581

EID cannot ensure accessibility for supplementary materials supplied by authors. Readers who have difficulty accessing supplementary content should contact the authors for assistance.

Portraying Tuberculosis Through Western Art, 1000–2000 CE

Appendix

Databases used in search strategy

France

- Atlas (Louvre): https://collections.louvre.fr
- POP: https://www.pop.culture.gouv.fr
- Image d'art: https://art.rmngp.fr/fr
- PhoCem: http:// www2.culture.gouv.fr/documentation/phocem/accueil.htm
- AGORHA: https://agorha.inha.fr/explorer-les-bases

Belgium

- Base ARTémis, collections de la Fédération Wallonie-Bruxelles
- Collections publiques de Flandres (Gand, Anvers, Bruges)
- Bruxelles, musées royaux des Beaux-Arts
- Anvers, musée royal des Beaux-Arts
- Anvers, collections des musées de la ville

The Netherlands

- Base des collections publiques des Pays-Bas
- Banque de données du RKD
- Base Ecartico de l'Université d'Amsterdam
- Biografisch portaal van nederland
- Monographie de l'Oranjezaal, par le RKD

- Amsterdam, Rijksmuseum
- Delft, Museum Het Prinsenhof
- Dordrecht, Dordrechts Museum
- Haarlem, Frans Hals Museum
- La Haye, Mauritshuis
- Leyde, Museum De Lakenhal
- Rotterdam, musée Boijmans Van Beuningen
- Utrecht, Centraalmuseum

Germany

- Deutsche Fotothek
- Portail d'images du BPK Bildagentur
- Collections publiques de Basse-Saxe
- Museen Nord, collections des musées de Schlesvig-Holstein
- Fondation des châteaux et jardins de Prusse
- Plastik im Mitteldeutschland (sculpture en Allemagne centrale), par l'Université de Halle
- Marbourg, Philips Universität, Bildarchiv Foto (photothèque de l'Université de Marbourg)
- Berlin, Staatliche Museen
- Brême, Kunsthalle
- Brunswick, Herzog Anton Ulrich Museum, et Wolfenbüttel, Herzog August Bibliothek, fonds d'art graphique
- Cassel, Gemäldegalerie Alte meister
- Dresde, Staatliche Kunstasmmlungen
- Düsseldorf, Kunstpalast

- Gotha, Schloss Friedenstein
- Hambourg, Kunsthalle
- Hambourg, Museum für Kunst und Gewerbe
- Karlsruhe, Staatliche Kunsthalle
- Munich, Alte Pinakothek
- Nuremberg, Germanisches Nationalmuseum
- Potsdam, Potsdam-Museum
- Stuttgart, Staatsgalerie
- Worms, Museum Heylshof

Austria

- Vienne, Kunsthistorisches Museum
- Vienne, Belvedere

Russia

- Moscou, musée Pouchkine, peintures italiennes
- Moscou, musée Pouchkine, art européen et américain du XIXe siècle
- Moscou, Musées du Kremlin
- Saint-Pétersbourg, musée de l'Ermitage

Switzerland

- Bâle, Kunstmuseum
- Coire, musée d'Art des Grisons
- Fribourg, musée d'Art et d'Histoire
- Genève, musée d'Art et d'Histoire
- Neuchâtel, musée d'Art et d'Histoire

Scandinavian countries

- Copenhague, Statens Museum for Kunst

- Oslo, Nasjonalmuseet
- Oslo, Munch Museet, catalogue des dessins d’Edward Munch
- Stockholm, Nationalmuseum
- Stockholm, Hallwylska Museum

Spain and Portugal

- Tapisseries flamandes en Espagne, par la Carlos Amberes Foundation
- Bilbao, Museo de Bellas Artes
- Madrid, musée du Prado
- Madrid, musée Thyssen-Bornemisza
- Valence, Museu de Belles Arts
- MatrizNet, patrimoine national portugais

Italy

- Archives photographiques de l’agence Fratelli Alinari, Florence
- Fondation Federico Zeri, Université de Bologne, photothèque
- Agence Scala Archives
- Manus, base des manuscrits conservés dans les bibliothèques publiques italiennes
- Florence, Galerie des Offices
- Gênes, Musei di Strada Nuova :
  - Palazzo Rosso
  - Palazzo Bianco
- Milan, Pinacothèque de Brera
- Milan, musée Poldi-Pezzoli
- Rome, musées du Vatican
- Rome, musées du Capitole
- Rome, Galleria Nazionale d’Arte Antica, Palazzo Barberini

- Rome, Accademia Nazionale di San Luca
- Rome, Villa Médicis, Académie de France à Rome (Base d'Antin)
- Turin, musées royaux
- Venise, Galleria dell'Accademia

UK and Ireland

- Archives photographiques de la Bridgeman Art Library
- Base « Art in UK »
- vads – The National Inventory of Continental European Paintings (base des peintures européennes non britanniques dans les collections britanniques)
- Government Art Collection, collections à la disposition des bâtiments publics britanniques
- National Trust
- Collections de la monarchie britannique
- Collections des musées nationaux écossais
- Barnard Castle, Bowes Museum
- Belfast, National Museum
- Birmingham, Museums and Art Gallery (collections préraphaélites)
- Bradford, National Science and Media Museum
- Cambridge, Fitzwilliam Museum
- Cardiff, National Museum of Wales
- Coventry, Base des collections des musées de Coventry
- Edimbourg, National Gallery of Scotland
- Glasgow, Collections des musées de la ville
- Glasgow, The Hunterian Museum and Art Gallery
- Liverpool, Walker Art Gallery

• Londres, National Gallery

• Londres, National Portrait Gallery

• Londres, Victoria & Albert Museum

• Londres, Tate Gallery

• Londres, The Wallace Collection

• Londres, Courtauld Institute

• Londres, Dulwich Picture Gallery

• Londres, Wellcome Collection

• Manchester, City Art Gallery

• Oxford, Ashmolean Museum of Art and Archeology

• Sheffield, Base des musées de Sheffield

• York, Museums and Gallery Trust

• Dublin, National Gallery of Ireland

Other European countries

• Collections du musée des Beaux-Arts de Budapest

• Varsovie, musée national

• Manuscriptorium, Bibliothèque numérique de la bibliothèque nationale de République tchèque

• Portail des musées d'Estonie

• Erevan, Galerie nationale d'Arménie

• Tbilissi, musée national géorgien

United States

• Art Resource

• DPLA, Digital Public Library of America

· Cambridge (Massachusetts), Harvard Library, Hollis images

· Répertoire de sculptures françaises dans les collections américaines

· (Pennsylvania), Bryan Mawr College; Haveford College; Swarthmore College

· Ann Arbor (Michigan), University of Michigan, Museum of Art

· Atlanta (Georgia), High Museum of Art

· Austin (Texas), Blanton Museum of Art

· Baltimore (Maryland), The Walters Art Museum

• Baltimore (Maryland), Museum of Art

• Berkeley (California), Berkeley Art Museum and Pacific Film Archive

• Birmingham (Alabama), Museum of Art

• Bloomfield Hills (Michigan), Cranbrook Art Museum

• Bloomington (Indiana), Indiana University, The Eskenazi Museum of Art

• Boston (Massachusetts), Museum of Fine Arts

• Boston (Massachusetts), Isabella Stewart Gardner Museum

• Brunswick (Maine), Bowdoin College Museum of Art

• Buffalo (New York), Albright Knox Art Gallery

• Cambridge (Massachusetts), Harvard Art Museums

• Champaign (Illinois), University of Illinois, Krannert Art Museum

• Chapel Hill (North Carolina), The University of North Carolina, Ackland Art Museum

• Charlottesville (Virginia), The Fralin Museum of Art

• Chicago (Illinois), Art Institute

• Chicago (Illinois), Smart Museum of Art

• Cincinnati (Ohio), Art Museum

• Cleveland (Ohio), The Cleveland Museum of Art

• Columbus (Ohio), Museum of Art

• Corning (New York), Corning Museum of Glass

• Dallas (Texas), Meadows Museum

• Dallas (Texas), Museum of Art

• Five Colleges and Historic Deerfield Museum Consortium (Massachusetts)

• Denver (Colorado), Art Museum

• Des Moines (Iowa), Salisbury House and Gardens

• Detroit (Michigan), Institute of Arts

• Durham (North Carolina), The Nasher Museum of Art at Duke University

• Eugene (Oregon), University of Oregon, Jordan Schnitzer Museum of Art

• Fort Worth (Texas), Kimbell Art Museum

• Glens Falls (New York), The Hyde Collection

• Greenville (North Carolina), Greenville Museum of Art

• Hagerstown (Maryland), Washington County Museum of Fine Arts

• Hanover (New Hampshire), Dartmouth College, Hood Museum of Art

• Hartford (Connecticut), Wadsworth Atheneum

• Honolulu (Hawaii), Museum of Art

• Houston (Texas), The Museum of Fine Arts

• Indianapolis (Indiana), Museum of Art

• Ithaca (New York), Cornell University, Herbert F. Johnson Museum of Art

• Kansas City (Missouri), The Nelson-Atkins Museum of Art

• Lawrence (Kansas), The University of Kansas, Spencer Museum of Art

• Little Rock (Arkansas), Arkansas Arts Center

• Los Angeles (California), County Museum of Art

- Los Angeles (California), The Jean-Paul Getty Museum of Art
- Madison (Wisconsin), Chazen Museum of Art
- Manchester (New Hampshire), Currier Museum of Art
- Memphis (Tennessee), Brooks Museum of Art
- Miami (Florida), Florida International University, Patricia and Philip Frost Art Museum
- Milwaukee (Wisconsin), Art Museum
- Milwaukee (Wisconsin), Marquette University, Haggerty Museum of Art
- Minneapolis (Minnesota), Institute of Arts
- Montgomery (Alabama), Museum of Fine Arts
- New Haven (Connecticut), Yale University Art Gallery
- New-York (New York), Dahesh Museum of Art
- New-York (New York), The Metropolitan Museum of Art
- New-York (New York), Corsair – The Morgan Library and Museum
- New-York (New York), The Frick Collection
- New-York (New York), Brooklyn Museum
- New-York (New York), Queens College, Godwin-Ternbach Museum
- Norfolk (Virginia), Chrysler Museum of Art
- New Orleans (Louisiana), Museum of Art
- Muncie (Indiana), Ball State University, David Owsley Museum of Art
- Oberlin (Ohio), Oberlin College, Allen Memorial Art Museum
- Pasadena (California), The Norton Simon Museum
- Philadelphia (Pennsylvania), Museum of Art
- Philadelphia (Pennsylvania), The Barnes Foundation

- Philadelphia (Pennsylvania), La Salle University Art Museum
- Pittsburgh (Pennsylvania), Carnegie Museum of Art
- Pittsburgh (Pennsylvania), The Frick Art Museum
- Portland (Oregon), Art Museum
- Poughkeepsie (New York), The Frances Lehman Loeb Art Center
- Princeton (New Jersey), University Art Museum
- Providence (Rhode Island), Rhode Island School of Design Museum
- Provo (Utah), The Brigham Young University Museum of Art
- Raleigh (North Carolina), North Carolina Museum of Art
- Richmond (Virginia), Virginia Museum of Fine Art
- Rochester (New York), Rochester University, Memorial Art Gallery
- Saint-Louis (Missouri), Art Museum
- Salem (Oregon), Hallie Ford Museum of Art, Willamette University
- Salt Lake City (Utah), Utah Museum of Fine Arts
- San Antonio (Texas), McNay Art Museum
- San Diego (California), Museum of Art
- San Francisco (California), Fine Arts Museums
- San Marino (California), The Huntington Library, Art Collections, and Botanical Gardens
- Santa Fe (New Mexico), New Mexico Museum of Art
- Sarasota (Florida), The John & Mable Ringling Museum of Art
- Seattle (Washington), Art Museum
- Seattle (Washington), Frye Art Museum
- Springville (Utah), Museum of Art

- Stanford (California), Cantor Arts Center
- Syracuse (New York), University Art Galleries
- Toledo (Ohio), Museum of Art
- Tulsa (Oklahoma), The Philbrook Museum of Art
- University Park (Pennsylvania), The Pennsylvania State University, Palmer Museum of Art
- Washington (District of Columbia), The National Gallery of Art
- Washington (District of Columbia), Hillwood Museum
- Washington (District of Columbia), Smithsonian Institution
- Wellesley (Massachusetts), Wellesley College, Davis Museum
- Williamsburg (Virginia), College of William and Mary, Muscarelle Museum of Art
- Williamstown (Massachusetts), The Sterling & Francine Clark Art Institute
- Worcester (Massachusetts), Art Museum

Canada

- Base Artefacts Canada
- Province du Québec, répertoire culturel
- Kingston (Ontario), Agnes Etherington Art Centre
- Montréal, musée des Beaux-Arts
- Ottawa, National Gallery of Canada
- Québec, musée national des Beaux-Arts
- Saint-Jean, musée du Nouveau-Brunswick
- Toronto, Art Gallery of Ontario
- Winnipeg, Art Gallery
